# Editorial: IL-1 Family Members in Health and Disease

**DOI:** 10.3389/fimmu.2019.02596

**Published:** 2019-11-13

**Authors:** Elizabeth Brint, Thomas Kamradt, Sarah L. Doyle

**Affiliations:** ^1^Department of Pathology, University College Cork, Cork, Ireland; ^2^APC Microbiome Ireland, Cork, Ireland; ^3^Institute of Immunology, University Hospital - Friedrich Schiller University Jena, Jena, Germany; ^4^Department Clinical Medicine, School of Medicine, Trinity College Dublin, Dublin, Ireland; ^5^Trinity Institute of Neuroscience, Trinity College Dublin, Dublin, Ireland

**Keywords:** inflammation, disease, interleukin, IL-1, IL-18, IL-33

In 1985, two distinct cDNAs encoding proteins sharing human Interleukin-1 (IL-1) activity were described, thus defining the first two members of the IL-1 family—IL-1α and IL-1β. These potent pro-inflammatory cytokines have been the subject of much research in the area of fever and inflammation, as well as for their roles in a myriad of inflammatory associated diseases. Over the years the family expanded to include cytokines with both pro- and anti- inflammatory properties, including IL-18, IL-33, IL-36, IL-37, IL-38, IL-1 receptor antagonist (IL-1Ra), and IL-36Ra. In addition to the 11 identified cytokine members of this family, there are now multiple discrete receptor family members, that form 4 functional receptor complexes able to activate downstream signaling cascades, as well as several decoy and inhibitory receptors.

The inflammatory functions of these cytokines have been well-defined for the long established family members but, in the case of some of the newer members such as IL-36, IL-37, and IL-38, their physiological roles are still being elucidated. For the pro-inflammatory cytokines, their primary functions involve activating signaling cascades and mediating the inflammatory response to a wide variety of signals, thereby co-ordinating innate and adaptive immune responses. To achieve this, the IL-1 family cytokine binds to its cognate receptor which, in the case of all family members, then causes recruitment of a specific accessory receptor. This interaction then allows for recruitment of the signaling adaptor protein MyD88, the common adaptor to all family members. MyD88 engagement to the receptor complex subsequently results in the activation of the downstream kinases, the IRAK family of proteins. Ultimately, through recruitment and activation of additional signaling intermediates, key transcription factors, such as AP-1 and NFκB, become activated translocate to the nucleus and result in transcription of a myriad of immune and inflammatory genes. The importance of these protein:protein interactions and this pathway has long been understood to hold the key to regulation of inflammation by IL-1 family members.

As is well-known, however “with great power comes great responsibility.” Therefore, given the ability of IL-1 family members to so potently drive and upregulate the inflammatory response, it is possibly not surprising that in recent years many members of this family have been identified as being critical for the development of diverse inflammatory and allergic diseases with much work focusing on IL-1 itself. Elevated plasma levels of IL-1, together with data from *in vitro* studies and murine models, have resulted in an association of IL-1 with an array of auto-inflammatory and autoimmune diseases. Perhaps the most illuminating data on the importance of IL-1 in the exacerbation of disease states has been gained from studies inhibiting the mechanism of action of these cytokines. Autoinflammatory conditions such as familial Mediterranean fever are characterized by recurrent bouts of fever in conjunction with debilitating local and systemic inflammation. These have proven to be responsive to IL-1β inhibition following administration of the naturally occurring IL-1 Receptor antagonist (IL-1Ra). Similarly, administration of IL-1Ra (now known by its generic name of anakinra) or administration of mAbs developed to target IL-1beta such as canikinumab, have also exhibited therapeutic efficacy in a range of diseases including type-2 diabetes, gout and rheumatoid arthritis. Indeed anakinra is now an approved therapy for the auto-inflammatory condition, Cryopyrin-Associated Periodic Syndromes and the autoimmune disease Rheumatoid arthritis, whilst canakinumab is an approved therapy for numerous auto-inflammatory conditions. It seems likely that in the coming years these therapies will be extended as approved treatments for additional diseases, as the therapeutic efficacy of targeting IL-1 in diseases ranging from neovascular disease to cancer is now being demonstrated in clinical trials. Indeed, as is highlighted by the recent findings from the CANTOS trial, IL-1β inhibition provides protection from the development of lung cancer.

The therapeutic potential of these family members is not solely limited to IL-1. IL-18 has been linked with a multitude of disease pathologies from retinal neovascular disease to inflammatory bowel disease. In terms of therapeutic targeting of IL-18, there is a current emphasis on inhibition of IL-18 function in type 2 diabetes, whilst the therapeutic benefit of activating IL-18 is being explored for both cancer and neovascular retinal disease. The function of IL-33, was first comprehensively explored in Th2 cells and asthma, given the high level of expression of its receptor in these cells, but has since been linked to other pathological conditions. To date however, therapeutic manipulation of IL-33 is still mainly centered around its role in asthma and allergic disease, with recent reports from a phase 2 clinical trial run by Regeneron and Sanofi demonstrating positive results for treatment of asthma with inhibition of IL-33. Like IL-1, IL-18, and IL-33, the more recently characterized IL-36 similarly plays a role in a variety of inflammatory diseases. Over activity of IL-36, has been implicated in psoriasis with negative outcomes. In contrast, administration of IL-36 in murine cancer models appears to be efficacious in the reduction of tumor growth. Phase 1 clinical trials targeting IL-36 signaling in a rare form of psoriasis (general pustular psoriasis) have shown positive results with this compound now progressing to phase 2. The role of IL-37 is the least understood with both pro and anti-inflammatory functions assigned to it.

In fact, opposing functions for many in this cytokine family have been shown, and as such it is clear that caution will have to be exercised with respect to the ongoing development of drugs targeting this family. Whereas, both IL-18 and IL-36 have demonstrated clear roles in the pathogenesis of certain auto-immune conditions, both have been seen to play a positive role in cancer. Additionally, both IL-1 α and IL-33 function as extracellular cytokines and as nuclear transcription factors, with their roles in the nucleus still poorly understood, Targeting of these cytokines therefore, may cause significant unwanted by-stander effects meaning that drug development will need to be nuanced to avoid unwanted interference in homeostatic roles. Therefore, while this family of cytokines appears to present excellent targets for many diseases, continued study and a deeper understanding of the pleiotropy of their functions is required. The substantial impact that drugs targeting this family of cytokines have the potential to provide for human health is evidenced by the sheer breadth of disease types that IL-1 family cytokines regulate. This is highlighted in this special topic and demonstrates that drugs developed to therapeutically manipulate this family of cytokines have the potential to cast a very wide net.

## Author Contributions

All authors listed have made a substantial, direct and intellectual contribution to the work, and approved it for publication.

### Conflict of Interest

The authors declare that the research was conducted in the absence of any commercial or financial relationships that could be construed as a potential conflict of interest.

